# The effects of LncRNA PVT1 on clinical characteristics and survival in breast cancer patients: A protocol for systematic review and meta analysis

**DOI:** 10.1097/MD.0000000000024774

**Published:** 2021-02-26

**Authors:** Li Xie, Gang Feng, Ping Zhu, Jiang Xie

**Affiliations:** a Department of Thyroid Breast Surgery; b Department of Nephrology, The First College of Clinical Medical Science, ChinaThree Gorges University, Yichang Central People's Hospital, Yichang; c Department of Hepatological surgery, China Resources Wisco General Hospital, Wuhan, China.

**Keywords:** breast cancer, long noncoding RNAs, meta-analysis, plasmacytoma variant translocation 1, prognosis

## Abstract

**Background:**

Currently, an increasing number of long noncoding RNAs (LncRNAs) have been reported to be abnormally expressed in human carcinomas and play a vital role in tumourigenesis. Some studies were carried out to investigate the influence of the expression of plasmacytoma variant translocation 1 (PVT1) on prognosis and its clinical significance in patients with breast cancer, while the results were contradictory and uncertain. A meta-analysis was conducted with controversial data to accurately assess the issue.

**Methods:**

A detailed search of relevant researches was performed in Wanfang, Chinese Biomedical Literature Database, Chinese National Knowledge Infrastructure, Chongqing VIP Chinese Science and Technology Periodical Database, PubMed, Embase, and Web of Science. Two reviewers independently conducted data extraction and literature quality evaluation. Odd ratio and its 95% confidence intervals were applied to evaluate the relationship between PVT1 and clinicopathological characteristics of breast cancer patients. Hazard ratios and its 95% confidence intervals were adopted to assess the prognostic effects of PVT1 on overall survival and disease-free survival. Meta-analysis was conducted with Stata 14.0 software

**Results:**

This study will provide high-quality evidence-based medical evidence for the correlation between PVT1 expression and overall survival, and disease-free survival and clinicopathological features.

**Conclusion:**

The study will provide updated evidence to evaluate whether the expression of PVT1 is in association with poor prognosis in patients with breast cancer.

**OSF REGISTRATION NUMBER:**

DOI 10.17605/OSF.IO/C2TYE.

## 1 Introduction

As the second leading cause of cancer-related death in women, breast cancer is the most common malignant tumor in women.^[1,2]^ Due to the lack of specific tumor markers, imaging and traditional serum markers, it is difficult to timely and effectively monitor the occurrence and development of tumors and the occurrence of drug resistance, thus resulting in poor clinical treatment. Early diagnosis and early treatment can improve the efficiency of clinical treatment, and it is especially important to improve the survival rate and life quality of patients. At present, there is no ideal biomarker for effective screening or diagnosis of cancer, which makes it urgent to find a new biological target that can guide carcinogenesis to detect cancer and predict survival.

Being a kind of RNA, Long noncoding RNAs (LncRNAs) are more than 200 bp long and lacks open reading frame and protein coding ability.^[3]^ Recent reports displayed that LncRNAs plays a vital role in the occurrence and development of cancer.^[4]^ With the application of high-throughput RNA sequencing technology, a large number of LncRNAs have been discovered.^[5]^ More and more evidence indicated that LncRNAs plays multiple and key roles in the occurrence and development of various cancers and non-cancerous diseases.^[6–9]^

LncRNA PVT1 is transcribed from PVT1 gene at 57 kb downstream of Myc gene, and deviate from the in-depth study to some extent for its complex structure and non-protein coding. With the epigenetic, transcriptional, and post-transcriptional levels involved in tumorigenesis and development, it was obvious that LncRNA plays a key role in human life activities, and then the study on LncRNA PVT1 has attracted people's attention again. Recent evidence suggested that PVT1 is abnormally regulated in a variety of human cancers.^[10–13]^ PVT1 participates in the proliferation, migration and invasion of tumor cells through various mechanisms.^[14]^ In addition, the overexpression of PVT1 is associated with the clinicopathological features of various cancers with high levels of PVT1, which suggest poor overall survival (OS) and disease-free survival (DFS).^[7,9,14,15]^ Therefore, more and more attention has been attached to the clinical application of PVT1 as a potential biomarker and therapeutic target for a variety of cancers.

A number of studies revealed that the high expression of PVT1 is closely related to the survival of patients suffering from breast cancer, but the results are also quite different.^[16,17]^ In order to more accurately analyze the impact of high expression of PVT1 on the survival of patients with breast cancer, this study comprehensively searched literatures that are related to the expression of PVT1 and the prognosis of patients with breast cancer, and adopted meta-analysis to evaluate the effects of high expression of PVT1 on the prognosis of patients with breast cancer.

## 2 Methods

### 2.1 Study registration

The protocol of the systematic review was registered on Open Science Framework, and the registration number is DOI 10.17605/OSF.IO/C2TYE. This meta-analysis protocol was based on the Preferred Reporting Items for Systematic Reviews and meta-analysis Protocols statement guidelines.^[18]^

### 2.2 Data sources and search strategy

Wanfang, Chinese Biomedical Literature Database, Chinese National Knowledge Infrastructure, the Chongqing VIP Chinese Science and Technology Periodical Database, PubMed, Embase, and Web of Science will be our electronic databases for retrieval. The retrieval time is from their inception to December 2020. The retrieval strategy will be created based on the discussion by all the researchers and the Cochrane handbook guidelines. The search strategy for PubMed is listed in Table 1. According to the actual situation of other electronic databases, the retrieval strategy can be modified.

**Table 1:** Search strategy in PubMed database.

Number	Search terms
#1	Breast Neoplasms[MeSH]
#2	Breast Cancer[Title/Abstract]
#3	Breast Tumors[Title/Abstract]
#4	Cancer of Breast[Title/Abstract]
#5	Cancer of the Breast[Title/Abstract]
#6	Human Mammary Carcinoma[Title/Abstract]
#7	Mammary Carcinoma, Human[Title/Abstract]
#8	Mammary Neoplasm, Human[Title/Abstract]
#9	Mammary Neoplasms, Human[Title/Abstract]
#10	Neoplasms, Breast[Title/Abstract]
#11	Tumors, Breast[Title/Abstract]
#12	Breast Neoplasm[Title/Abstract]
#13	Breast Tumor[Title/Abstract]
#14	Cancer, Breast[Title/Abstract]
#15	Carcinoma, Human Mammary[Title/Abstract]
#16	Carcinomas, Human Mammary[Title/Abstract]
#17	Human Mammary Carcinomas[Title/Abstract]
#18	Human Mammary Neoplasm[Title/Abstract]
#19	Human Mammary Neoplasms[Title/Abstract]
#20	Mammary Carcinomas, Human[Title/Abstract]
#21	Neoplasm, Breast[Title/Abstract]
#22	Neoplasm, Human Mammary[Title/Abstract]
#23	Neoplasms, Human Mammary[Title/Abstract]
#24	Tumor, Breast[Title/Abstract]
#25	or/1–24
#26	Plasmacytoma variant translocation 1[Title/Abstract]
#27	PVT1[Title/Abstract]
#28	or/26–27
#29	Prognos^∗^
#30	survival
#31	or/29–30
#32	#25 and #28 and #31

### 2.3 Inclusion criteria for study selection

The included articles must meet the following inclusion criteria:

(1) Patients who were diagnosed with breast cancer based on pathology and histology. No restrictions on region, race, or age.
(2) The expression of PVT1 in related breast cancer tissues.
(3) Reported PVT1 survival-related data, including OS and DFS.
(4) Patients are divided into PVT1 positive (high) and PVT1 negative (low).
(5) The relationship between clinicopathological parameters and prognosis of PVT1 was introduced in details.
(6) Published as full-text articles, original Chinese and English Research papers.

The criteria for excluding literature are summarized as follows:

(1) Relevant critical articles or conference articles and related case reports or replying emails.
(2) Documents that cannot be tested in electronic databases.
(3) Non-human experiments were carried out.
(4) It was impossible to accurately express PVT1 and count the clinical characteristics and overall survival data of breast cancer.
(5) Repeatedly published literature.

### 2.4 Data collection and analysis

#### 2.4.1 Selection of studies

**Figure 1:**
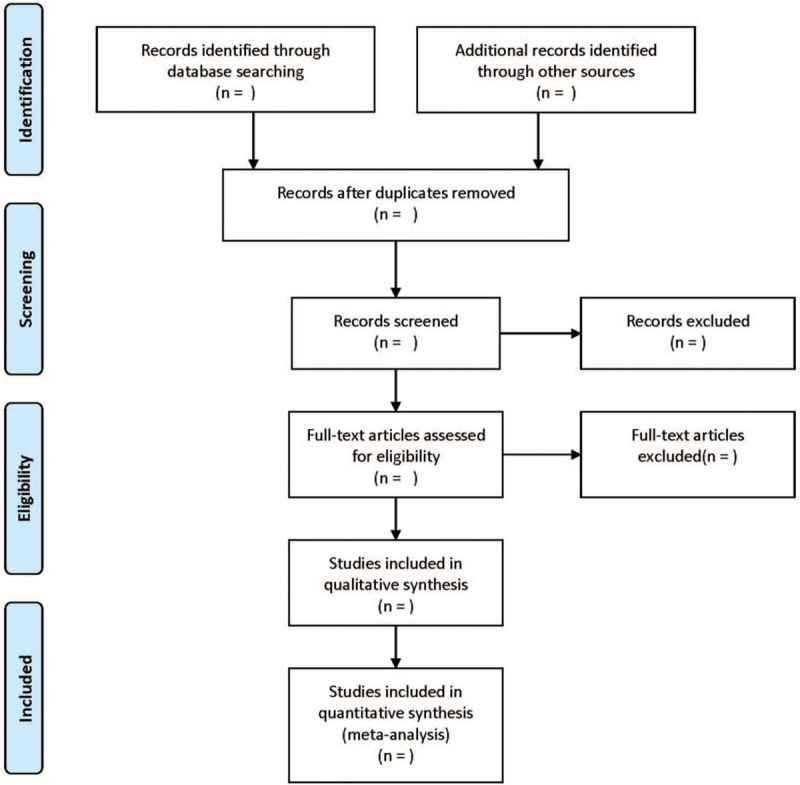
Flow diagram of study selection process.

According to the Preferred Reporting Items for Systematic Reviews and MetaAnalysis flowchart (Fig. 1), all reviewers received evidence-based training and adhered to the process summarized. The 2 reviewers independently screened the literature based on the title, abstract and key words of literatures, and excluded the irrelevant literatures. The rest of literatures were further confirmed by 2 researchers after reading the full text. The excluded research and the reasons for the exclusion were recorded. The differences between the 2 reviewers were resolved through consensus or a third independent reviewers.

#### 2.4.2 Data extraction and management

According to the inclusion and exclusion criteria, 2 reviewers independently extracted data from the eligible studies. Data was double input to EpiData software (version 3.0; The EpiData Association, Odense, Denmark) by 2 reviewers. Differences were resolved through group discussions. The extracted information includes the first author name, publication year, ethnicity, total patient number, detection method for PVT1 expression, number of patients in high and low PVT1 expression groups, number of patients with lymph node melanoma metastasis (LNM), tumor-node-metastasis (TNM) stage, lymphatic invasion, vascular invasion, invasion depth in high and low PVT1 expression groups, survival analysis method, cut-off value to define the high and low PVT1 expression, HRs and corresponding 95% CIs for OS, and DFS, if provided. If the data was analyzed by both univariate and multivariate methods, the latter was preferred. We obtained hazard ratios (HRs) and confidence intervals (95%CIs) from the Kaplan–Meier survival curves by using Engauge Digitizer version 4.1 (http://digitizer.sourceforge.net/).

### 2.5 Assessment of quality in included studies

The quality of all the included studies will be evaluated by 2 reviewers independently based on the Newcastle–Ottawa scale (NOS) that is used to evaluate the quality of observational studies.^[19]^ Disagreement will be reported and resolved by a third reviewer. Three broad perspectives of each study quality will be scored: the selection of the study groups, the comparability of the case and control groups, and the determination of the exposure or outcome of interest in the studies. The NOS values arrange from 0 to 9. Studies with a score of 6 are considered to be of high quality.^[20]^

### 2.6 Measures of prognosis

OS and DFS would be taken as prognostic outcomes. The results would be expressed as HRs with 95% CIs.

### 2.7 Management of missing data

If there is insufficient or missing data in the literature, we would contact the author via email to request the data. If the data is not available, we would only analyze currently available data and discuss its potential impact.

### 2.8 Statistical analysis

Statistical analysis was performed using STATA 14.0 (STATA Corporation, College Station, TX). The 95% CIs and HRs were applied to evaluate the relationship between PVT1 expression and OS and DFS. Odds ratio and 95% CIs were used to evaluate the impact of PVT1 expression on clinicopathological characteristics. First, statistical heterogeneity tests were performed on the included studies. If there is no statistical heterogeneity among the included literatures (I2 < 50%, *P* ≥ .1), a fixed effect model is used. When there is statistical heterogeneity among the included literatures (*P* < .1, I2 > 50%), the sources of heterogeneity would be analyzed. Clinical heterogeneity would be treated by subgroup analysis. In the absence of significant clinical heterogeneity and methodological heterogeneity, statistical heterogeneity would be considered, and random effects models would be adopted for analysis. If the clinical heterogeneity of the subgroup analysis is significantly higher, no meta-analysis would be conducted, only a descriptive analysis.

### 2.9 Additional analysis

#### 2.9.1 Subgroup analysis

We will conduct a subgroup analysis based on the type of breast cancer, the detection method of PVT1 expression, race, and the source of survival data.

#### 2.9.2 Sensitivity analysis

The sensitivity analysis of each index was carried out by elimination method to check the stability of the results.

#### 2.9.3 Reporting bias

If the number of studies that are included in a certain outcome index is no less than 10, funnel chart is used to evaluate publication bias.^[21,22]^

### 2.10 Ethics and dissemination

The content of this article does not involve moral approval or ethical review and would be presented in print or at relevant conferences.

## 3 Discussion

In recent years, more and more studies proved that LncRNAs is abnormally expressed in various cancers, and some of them can even be used as potential markers for the diagnosis, prognosis, and treatment of malignant tumors. Therefore, a large number of reviews or meta-analysis articles, including our research, have been applied to reveal the relationship between LncRNAs and cancer progression and prognosis. PVT1 gene was first discovered in mice in 1983 and is often associated with plasmacytoma.^[23,24]^ Shortly after that, the PVT1 locus also appeared as a variant translocation site in human Burkitt lymphoma.^[25]^ Since then, as reflected in the increase in terms of the number of PVT1-related publications, PVT1 has been a focus area, especially in recent decades. Many studies have exhibited the overexpression of PVT1 in various cancer tissues and cell lines, and showed that the expression of PVT1 was negatively correlated with the prognosis of patients.^[26–29]^

The researchers found that PVT1 is significantly overexpressed in breast cancer and can be used as an independent predictor of survival and prognosis.^[16,17]^ The overexpression of PVT1 was significantly related to tumor size, pathological grade, Ki-67 positive rate and poor prognosis. In addition, after inhibiting the expression of PVT1, the proliferation, migration and invasion of breast cancer cells were inhibited by affecting the expression of p21^[17]^ that is not only the main molecule regulating cell cycle, but also cyclin-dependent kinase, and can inhibit the activity of cyclin kinase complex by binding to cyclin-dependent kinase complex to regulate G, restriction point and G1max S checkpoint. Wang et al^[16]^ verified that sOx2 can activate the expression of PVT1, and then promote the proliferation and metastasis of breast cancer through EMT On the contrary, knocking down PVT1 or blocking SOX2 can inhibit EMT and decrease the ability of tumor cells to invade and metastasize. These results suggested that PVT1 may play an important role in the occurrence and development of breast distemper and may be a target for the treatment of breast cancer. Therefore, we hope that this meta-analysis can provide more accurate and objective evidence for the relationship between PVT1 expression and prognosis in patients with breast cancer.

Our research includes some limitations. First of all, the detection methods, and thresholds of PVT1 expression may be different. Furthermore, there are a variety of treatment methods for patients, including surgery, chemotherapy, radiotherapy, targeted therapy, and immunotherapy and so on. Therefore, there may be a risk of heterogeneity Most importantly, this study only includes studies published in English and Chinese, so significant studies or reports may be omitted.

## Author contributions

**Conceptualization:** Li Xie, Ping Zhu.

**Data curation:** Li Xie, Ping Zhu, Gang Feng.

**Formal analysis:** Gang Feng and Ping Zhu.

**Funding acquisition:** Jiang Xie.

**Methodology:** Gang Feng and Ping Zhu.

**Project administration:** Jiang Xie.

**Resources:** Gang Feng.

**Software:** Gang Feng.

**Supervision:** Jiang Xie.

**Validation:** Li Xie.

**Visualization and software:** Li Xie.

**Writing – original draft:** Li Xie and Jiang Xie.

**Writing – review & editing:** Li Xie and Jiang Xie.
